# Functional outcomes with handsewn versus stapled anastomoses in the treatment of ultralow rectal cancer

**DOI:** 10.1007/s13304-017-0507-z

**Published:** 2018-01-08

**Authors:** Lisa Ramage, Paul Mclean, Constantinos Simillis, Shengyang Qiu, Christos Kontovounisios, Emile Tan, Paris Tekkis

**Affiliations:** 1 Department of Surgery and Cancer, Chelsea and Westminster Hospital, Imperial College London NHS Trust, 369 Fulham Road, London, SW10 9NH UK; 2 Department of Colorectal Surgery, The Royal Marsden NHS Foundation Trust, London, UK; 3 Department of Colorectal Surgery, Singapore General Hospital, Singapore, Republic of Singapore

**Keywords:** Function, Coloanal anastomosis, Intersphincteric resection, Quality of life

## Abstract

Adequate oncological outcomes have been demonstrated with rectal resection and handsewn coloanal anastomosis (CAA) in tumours in close proximity to the internal anal sphincter. Our aim was to assess functional differences between handsewn CAA and ultralow stapled anastomosis. Participants were identified from a single-surgeon series. Included participants underwent anorectal physiology testing of anal sphincter function, in addition to completion of several questionnaires: Wexner Incontinence Score (WIS); Birmingham Bowel, Bladder and Urinary Symptom Questionnaire (BBUSQ); Low Anterior Resection Syndrome (LARS) Score; SF36. Non-parametric data compared using the Mann–Whitney *U* test. 20 participants were included; 11 stapled and 9 handsewn. Mean follow-up was 2.95 ± 1.97 years. The mean LARS score was 21.9 ± 1.97 years in the stapled group versus 29.4 ± 9.57 in the handsewn group (*p* = 0.133). The Wexner incontinence score was significantly higher in the handsewn group (*p* = 0.0076), with a mean score of 4.6 ± 3.69 versus 10.9 ± 4.76. The incontinence domain of the BBUSQ was also significantly worse in patients with a handsewn anastomosis (*p* = 0.001). With the exception of general health (*p* = 0.035) and social functioning (*p* = 0.035), which were worse in the handsewn groups, the other six domains of the SF-36 showed no statistical difference between groups. Anorectal physiology scores were not significantly different. Handsewn CAA anastomosis is known to be safe and oncologically feasible. Patient selection should be vigorous, with preoperative counseling regarding the likelihood of incontinence to manage patients’ expectations and promote comparable quality of life in the long-term.

## Introduction

Consensus as to the adequate distal clearance margin required to safely achieve oncological tumour clearance in bowel cancer resection has shifted from 5 cm [1] to 1 cm [2]. This has facilitated the performance of ultralow and intersphincteric resection (ISR) in cases where the tumour is in proximity to or involving the uppermost edge of the internal anal sphincter in patients who would have otherwise been left with a permanent colostomy. We have previously shown that comparable oncological outcomes can be achieved with handsewn coloanal anastomosis (CAA) [3]. With increasing emphasis being placed on long-term cancer survivorship, and the recognition that 5-year survival rates for rectal cancer patients are increasing, currently in the region of 60%, the importance of acceptable functional outcomes and quality of life is a key issue in treatment decisions.

The aim of this paper was to ascertain whether there were any differences in long-term functional outcomes following rectal resection with CAA compared to an ultralow stapled anastomosis through analysis of a single-surgeon–patient cohort.

## Methods

This observational study received a favourable opinion from the Northampton Research Ethics Committee (REC14/LO/1653). Patients were identified from a prospectively maintained database of all rectal cancer resections performed across multiple sites by a single consultant colorectal surgeon with extensive experience in resection of complex and recurrent rectal tumours. Patients may have had additional neoadjuvant chemoradiotherapy or adjuvant chemotherapy.

Patients were contacted and invited to attend for comprehensive functional assessment carried out within a tertiary referral centre for Pelvic Floor Dysfunction.

Inclusion criteria were as follows:

1. Disease-free and oncological treatment completed at least 6 months ago.
2. Any diverting stoma reversed at least 6 months ago.
3. Either performance of a handsewn (coloanal) anastomosis OR a stapled anastomosis with anastomotic height within 3 cm of the dentate line.

Exclusion criteria:

1. Active proctitis.
2. Permanent diverting stoma.
3. Recurrent or distant disease.

Participants who consented to study inclusion were invited to attend a one-off assessment which included patient interview, the completion of several validated patient questionnaires which aimed to assess functional outcomes, quality of life (QoL) data and postoperative sexual function:

1. Wexner Incontinence Score [4].
2. Birmingham Bowel, Bladder and Urinary Symptom Questionnaire, (BBUSQ) [5]: four domains: constipation, evacuation, faecal incontinence, urinary.
3. Low Anterior Resection Syndrome (LARS) Score [6].
4. Short-Form 36 (SF-) 36 [7].
5. Additionally, patients were asked to complete a sexual function score (Male Sexual Health Questionnaire, MSHQ [8], and Female Sexual Function Index, FSFI) [9], although completion of these was entirely voluntary.

Following from this, patients underwent physical examination, which included digital rectal examination (DRE) and anorectal physiology (ARP) assessment of motor and sensory function.

### Surgical methods

In all cases, a laparoscopic approach was performed where feasible. Surgical methods employed by the operating surgeon to create a handsewn anastomosis have previously been described [3]. Patients who underwent handsewn anastomosis had one of three slightly different approaches depending on the relation of the tumour to the dentate line, as per the classification outlined by Rullier et al. [10]. A straight coloanal anastomosis was performed in all cases.

### Anorectal physiology

Anorectal physiology was performed using T-DOC^®^ Air-Charged™ ARM Catheters. These have four pressure sensors measuring four quadrants of the anal sphincter, plus a distal balloon which is slowly filled to obtain sensory responses.

The pressure sensors were connected to the Delphis IP processor by Laborie Urodynamics, The procedure was carried out in the left lateral position. After DRE was performed to assess for any stricture or contraindications to insertion, the catheter was inserted into the anal canal.

#### Motor function assessment

The maximum resting pressure (in mmHg) was noted at the high-pressure zone (HPZ). The mean incremental squeeze pressure was calculated as an average of three squeeze readings taken at the level of the HPZ. The mean five-second incremental squeeze pressure calculated in a similar manner. Lastly, the mean cough squeeze increment was calculated.

#### Sensory function assessment

A balloon at the end of the catheter was slowly inflated with air whilst inside the rectum. Patients were asked to indicate the volume at which they were first aware of the balloon (threshold volume), then the volume at which they would usually consider opening their bowels (urge volume), before finally the maximal volume which they could tolerate (threshold volume).

### Statistical analysis

Non-parametric data were compared using Mann–Whitney *U* test. Data analysis was performed using SPSS software. A *p* value of < 0.05 was considered to be statistically significant.

## Results

### Study demographics

A total of 20 patients were recruited to this study over a 12 month period. There were 9 CAA versus 11 stapled anastomoses. The demographics of each group are shown in Table 1.

**Table 1:** Study demographics

	Handsewn (*n* = 9)	Stapled (*n* = 11)
Age*	66.5 (55–72)	57 (33–77)
M:F ratio	7:2	8:3
Postoperative staging	T0N0: 1 T1N0: 3 T2N0: 4 T2N2: 1	T2N0: 3 T2N1: 1 T3N0:3 T3N1: 2 T3N2a: 1
Preoperative radiotherapy	5	6
Neoadjuvant/adjuvant chemotherapy	4	8
Time surgery to assessment (years)*	3.62 (1.12–8.44)	2.04 (1.08–2.88)
Complications	3: anastomotic stricture 1: pelvic collection	2: anastomotic leak 1: pelvic collection
Anastomosis height	CAA 1: 2 CAA 2: 6 CAA 3: 1	3 cm^φ^: 5 2 cm: 2 1 cm: 3 <1 cm: 1
Operation type	Open: 8 Laparoscopic: 1	Open: 8 Laparoscopic: 3

CAA1—tumour > 2 cm from dentate line; anastomosis above dentate line with full preservation of internal anal sphincter. CAA2—tumour 1–2 cm from dentate line, partial excision of internal anal sphincter; CAA3—tumour involving/abutting uppermost internal anal sphincter, internal anal sphincter resection leaving 1 cm distal cuff

*Values are expressed as mean (range)

^φ^Cm from dentate line

Follow-up from date of surgery to date of assessment was at a mean of 2.95 years ± 1.97 years overall. One patient in the stapled versus three in the handsewn group required the use of rectal irrigation after restoration of normal bowel continuity. Three patients in the handsewn group required sacral nerve stimulation.

### Functional scores

The results of the LARS Score, Wexner Incontinence Score and Birmingham Bowel, Bladder and Urinary Symptom Questionnaire are shown in Table 2.

**Table 2:** Functional scores

	Handsewn	Stapled	*p* value
LARS Score	29.4 ± 9.57	21.9 ± 10.86	0.133
Wexner Incontinence Score	10.9 ± 4.76	4.6 +/03.69	0.0076
Birmingham Bowel, Bladder and Urinary Symptom Questionnaire			
Constipation domain	35.37 ± 19.03	41.0 ± 9.82	0.182
Evacuation domain	23.58 ± 10.65	16.19 ± 11.02	0.182
Faecal incontinence	53.70 ± 27.038	16.67 ± 11.79	0.001
Urinary domain	12.96 ± 10.04	12.61 ± 9.12	0.968

The mean LARS Score was 21.9 ± 10.86 in the stapled versus 29.4 ± 9.57 in the handsewn group, and was not statistically different between groups (*p* = 0.133).

The Wexner incontinence score was significantly higher in the handsewn group (*p* = 0.0076), with a mean score of 4.6 ± 3.69 versus 10.9 ± 4.76.

The Birmingham Bowel, Bladder and Urinary Symptom Questionnaire (BBUSQ) is scored from 0 to 100% for each of the four domains (constipation, evacuation, faecal incontinence and urinary), with a higher score indicating worse symptoms. Neither group had an “abnormal” score in the constipation domain (abnormal > 64%), with a mean of 35.37 ± 19.03 in the handsewn versus 41.0 ± 9.82 in the stapled group (*p* = 0.133). Similarly with the urinary domain, neither groups of patients had an abnormal score (> 20%), with a mean of 12.96 ± 10.04 in the handsewn versus 12.61 ± 9.12 in the stapled group (*p* = 0.968).

The faecal incontinence score was significantly worse in the handsewn group (53.7 ± 27.03) versus 16.67 ± 11.79 in the stapled group (*p* = 0.001); the stapled group had a score which was “normal” according to the validation criteria (< 17%).

The evacuation domain score was 23.58 ± 10.65 in the handsewn versus 16.19 ± 11.02 in the stapled group (*p* = 0.182). The score in the handsewn group was classified as “abnormal” according to the validation criteria cut-off (> 17%).

### Quality of life: SF-36 Score

The results across the eight domains of the SF-36 are shown in Table 3. Scores for each domain are out of 100%, with a lower score indicating worse QoL. The handsewn group had a worse score for each of the eight domains of the SF-36, however, this only reached statistical significance with general health (58.81 ± 22.19 versus 76.50 ± 14.90%, *p* = 0.035) and social functioning (63.89 ± 31.53 versus 91.25 ± 15.64%, *p* = 0.035).

**Table 3:** SF-36 Scores

SF-36 domain	Handsewn (*n* = 9)	Stapled (*n* = 10)	*p* value
Physical function	76.91 ± 27.2	92.39 ± 10.37	0.211
Role physical	65.28 ± 31.73	85.00 ± 21.08	0.211
Bodily pain	70.83 ± 27.95	87.50 ± 18.63	0.182
General health	56.81 ± 22.19	76.50 ± 14.90	0.035
Vitality	50.69 ± 19.38	62.50 ± 13.82	0.278
Social functioning	63.89 ± 31.53	91.25 ± 15.65	0.035
Role emotional	73.96 ± 28.33	88.33 ± 18.09	0.274
Mental health	62.22 ± 17.87	74.00 ± 7.38	0.156

### Anorectal physiology

Anorectal physiology results are displayed in Table 4. With regards to motor function, no significant difference was detected between groups with either resting or squeeze pressures. Both groups had a mean value for the motor pressures which were within normal parameters.

**Table 4:** Anorectal physiology results

	Handsewn	Stapled	*p* value
Motor			
Maximum resting pressure (mmHg)	45.11 ± 15.96	55.0 ± 12.10	0.299
Mean squeeze pressure increment (mmHg)	68.56 ± 60.78	105.39 ± 60.75	0.299
Mean five-second squeeze increment (mmHg)	82.33 ± 65.46	69.69 ± 39.76	1.00
Mean cough pressure increment (mmHg)	87.67 ± 62.46	92.65 ± 64.58	1.00
Sensory			
Threshold volume (ml)	16.75 ± 7.32	30.7 ± 28.20	0.397
Urge volume (ml)	36.33 ± 36.48	70.43 ± 65.24	0.051
Maximum tolerated volume (ml)	63.33 ± 72.43	117.86 ± 86.02	0.051

With regards to sensory function, the handsewn group had a lower threshold volume than the stapled, although this was not statistically significant (*p* = 0.397). The urge volume and maximum tolerated volumes were also reduced in the handsewn group (36 versus 70 ml and 63 versus 118 ml, respectively), both of which were approaching statistical significance.

### Sexual function

Sexual function data was only available for two males in the stapled group, and three males and two females in the handsewn group. All males scored poorly on the erection scale. Table 5 gives the results. The results of the Female Sexual Function Index are shown in Table 6.

**Table 5:** Male Sexual Health Questionnaire results

Patient no.	Group	Erection scale (max = 15)	Ejaculation scale (max = 35)	Satisfaction scale (max = 30)
1	Stapled	3	1	20
2	Stapled	4	32	16
3	Handsewn	3	10	15
4	Handsewn	0	21	18
5	Handsewn	0	15	23

Higher scores are indicative of better sexual function

**Table 6:** Female Sexual Function Index results

Patient no.	Group	Desire (max = 6)	Arousal (max = 6)	Lubrication (max = 6)	Orgasm (max = 6)	Satisfaction (max = 6)	Pain (max = 6)
1	Handsewn	6	1.2	1.2	1.2	ND	1.2
2	Handsewn	3.6	4.8	5.4	5.2	2.4	5.6

Higher scores are indicative of better sexual function

## Discussion

Intersphincteric resection with handsewn coloanal anastomosis is an alternative to extralevator abdominoperineal excision (ELAPE) with comparable oncological outcomes in addition to the perceived benefits of maintenance of bowel continuity and prevention of a stoma. Current recommendations set out by the MERCURY II study group indicate that where there is evidence of tumour extension on MRI beyond the muscularis propria or internal anal sphincter (IAS) and into the intersphincteric plane, then ELAPE should be performed [11]. Otherwise, ISR is considered to be a safe and valid alternative.

The technique of intersphincteric resection was first described by Lytle and Parks (1977), initially employed in resection of inflammatory bowel disease [12]. ISR in the context of rectal cancer was described by Braun et al. in 1992 [13], who reported a 62% 5-year survival rate versus 58% in ISR and abdominoperineal excision patients, respectively. 85% of patients had also reported good postoperative functional results. Rullier et al. [2] published a series of 92 patients in 2005 who underwent ISR, including 72 patients with T3 and 6 patients with T4 tumours, where there was evidence of invasion of the internal anal sphincter. Median distance of the tumour was 3 cm from the anal verge (range 1.5–4.5 cm). R0 resection was achieved in 89% of cases. They demonstrated a local recurrence rate of 2%, and distal recurrence of 19%. At 5 years, disease-free survival (DFS) was 70% and overall survival was 81%. They concluded that the use of adjuvant radiotherapy reduced positivity of circumferential margins and facilitated successful resection in more advanced tumours, and that ISR was acceptable in such situations.

Usually, ISR with handsewn coloanal anastomosis is reserved for cases either where there has been a failure of the stapling device, or the tumour is close to or involving the sphincter complex, where the procedure acts as an alternative to APER. In this situation, a cuff of proximal internal anal sphincter may be removed with the specimen to achieve the minimum 1 cm distal cut-off margin required for oncological curative resection to be achieved.

We have previously described three CAA techniques based on the level of the tumour [3] and related to the classification described by Rullier et al. [10]: type 1 CAA was performed above the dentate line, therefore not involving the internal anal sphincter, type 2 CAA was performed for tumours 1–2 cm from the denate line, and involved partial excision of the internal anal sphincter; type 3 CAA was performed for intra-anal tumours which were either abutting the IAS or involving the uppermost portion, and also involved partial IAS excision to leave a minimum of 1 cm of distal IAS. In a group of 71 patients, R0 resection was achieved in 97.1%, with the level of anastomosis having no relation to oncological outcomes. All patients had received an end-to-end straight anastomosis with no pouch formation.

The above results demonstrate a significant difference in both the Wexner Incontinence Score and the incontinence domain of the BBUSQ, with those having handsewn anastomosis having a worse functional outcome. Despite this, there was no significant difference found in six out of eight of the SF-36 domains. The handsewn group had a significantly worse score in the general health and social function domains compared to the stapled group, which may be at least partially explained by the higher risk of faecal incontinence episodes, which could impact, on confidence in social situations and may be a recurrent source of embarrassment for sufferers. The handsewn group did, however, have a lower mean score for each of the other six domains of the SF-36 when compared to the stapled group, and whilst this did not prove to be statistically significant, a larger sample size may have eluded to a more significant difference.

There are several studies which consider postoperative function with ISR. Bittorf et al. [14] discuss a cohort of 33 patients who underwent ISR and coloanal anastomosis at the level of the dentate line (2.9 ± 1.1 cm). 12/33 were given a colonic J pouch, the rest a straight coloanal anastomosis. 31/33 patients had closure of the diverting ileostomy. Of these, at least weekly incontinence of solid stool was experienced by 8/31 patients, and 17/31 for liquid stool and gas. 7/31 reported regular soilage. 24/31 patients reported pad usage at least weekly. 9 patients reported no change in deferral time, whereas 14 had impaired deferral and 8 were completely unable to defer defecation. Interestingly, 7/9 of those with no impairment of deferral time had been given a colonic J pouch. The postoperative Wexner Score was 12 ± 4.5, with the patients with a straight anastomosis reporting a significantly worse incontinence score (13.4 vs 9.9, *p* < 0.05). Chemoradiotherapy gave a significantly poorer Wexner Score (*p* = 0.054). Manometry results showed a reduction in the maximum and mean resting pressures when compared to healthy volunteers, with preserved squeeze pressure. Significantly worse tolerated volumes on sensory testing were seen with straight versus J pouch configurations, this was also the case with those who had undergone chemoradiotherapy.

Quality of life differences between low anterior resection (LAR), APER and intersphincteric resection (ISR) were considered by Konanz et al. [15] in a cohort comprised of 41 patients who underwent LAR, 33 had ISR and 50 APER. They found no significant differences between all three groups in terms of global quality of life, emotional, social and cognitive functioning. However, those undergoing LAR had higher physical functioning scores compared to APR patients (*p* = 0.0262), and APR patients had lower scores than ISR patients (*p* = 0.0280). ISR patients had significantly higher symptom scores with regards to defecatory disorders compared to those who had undergone LAR (*p* < 0.05); in addition, Wexner Incontinence Scores were higher in the ISR group (12.9 versus 9.5, *p* < 0.0005).

In a series recently published by Koyama et al. [16] comparing outcomes with 77 ISR patients, 68 LAR patient and 33 APER patients. Local recurrence rates were similar between all three groups. With regards to function, they found no difference in frequency of defecation between ISR and LARS patients. Urgency was experienced in 57% of ISR patients and 47% of LAR patients. Failure of discrimination was present in 11% of ISR versus 3% of LAR patients. These differences did not reach statistical significance. However, pad usage was significantly more common in ISR patients (84 versus 33%). The Wexner Incontinence Score was also significantly worse in ISR patients (8.1 versus 4.9 *p* = 0.004). Quality of life data was similar between groups.

Tokoro et al. [17] analysed results of 30 patients who underwent either partial, subtotal or total ISR (i.e. complete removal of internal anal sphincter) without adjuvant chemoradiation. In patients who had undergone partial ISR, postoperative Wexner Score significantly improved from 3 to 6 months postoperatively. By 6–12 months, there was a trend in those who had had a subtotal or total ISR towards improved function. At 3, 6 and 12 months, there was no significant differences between groups, however, those who did not suffer with concomitant stricture did have significant improvement of Wexner Scores from baseline compared from those who strictured. The presence of a stricture was also linked to ongoing nocturnal defecation and urgency, and worse outcomes at 12 months with regards to bowel frequency, fragmentation and urgency. Overall, the volume of residual internal anal sphincter was not found to affect bowel function.

Despite the increased risk of faecal incontinence with handsewn CAA, we have shown no significant differences in QoL in most domains. Overall, handsewn anastomosis has been shown to be safe and oncologically feasible compared to other techniques; however, it should be performed in specialist centres with experience of performing sphincter-preserving surgery. It is vital that patients are carefully selected for resection with CAA, taking into consideration factors such as patient age, any pre-existing functional deficit, the patient’s socioeconomic background and the potential need for any further adjuvant treatment. We would advocate full assessment of sphincter function preoperatively to include anorectal physiology and in women with a history of previous vaginal delivery, endoanal sphincter ultrasound to check sphincter integrity.
